# Program Director Perspectives on the Impact of the Proposed 48-Month Emergency Medicine Residency Requirement: A National Survey

**DOI:** 10.5811/westjem.48359

**Published:** 2025-11-26

**Authors:** Richard Austin, Chinmay Patel, Kristin Delfino, Sharon Kim

**Affiliations:** * Southern Illinois University, School of Medicine, Department of Emergency Medicine, Springfield, Illinois; † Baylor Scott & White All Saints Medical Center, Department of Emergency Medicine, Fort Worth, Texas; ‡ Southern Illinois University, School of Medicine, Department of Surgery, Springfield, Illinois

## Abstract

**Introduction:**

In early 2025, the Accreditation Council for Graduate Medical Education (ACGME) announced proposed revisions to emergency medicine (EM) residency training to include substantial changes to the length of training programs, required rotations, and structured experiences. To date, no published national survey has sought to determine how these changes would impact individual programs.

**Methods:**

Over a three-week period in April 2025, we anonymously surveyed program directors or their designees online through the Council of Residency Directors in Emergency Medicine listserv. Survey respondents were asked about the impact the changes would have on their programs and their overall opinions of the proposed 48-month minimum requirement.

**Results:**

A total of 86 program directors responded to the survey (response rate of 29.9%) with representative samples from current three-year (83.7%, 72/86) and four-year (16.3%, 14/86) programs. Most program directors reported that they would have to make significant revisions in either structured experiences, required rotations, or both. Most survey respondents from three-year programs (52/72) do not support the proposed changes, whereas all respondents from four-year programs (14/14) do support the changes (P<.001).

**Conclusion:**

Proposed program requirements may require modifications in both three- and four-year programs; 33 of the 86 program directors surveyed reported that would need more than one year to meet the requirements, if adopted. This raises the concern that programs may not be prepared to implement the revisions within the proposed timeline, potentially impacting resident education and the future EM workforce. The ACGME should consider a staged rollout of requirements to allow them to be thoughtfully implemented in a meaningful way.

## INTRODUCTION

On February 12, 2025, the Accreditation Council for Graduate Medical Education (ACGME) proposed significant revisions to the program requirements for emergency medicine (EM) residency training in the United States, with the most notable change being the standardization of training length to 48 months for all programs, effective July 1, 2027.^1^ This proposed change has generated considerable discussion and debate within the EM community, with concerns raised about its potential impact on resident education, program finances, and the EM workforce. Currently, most programs are three years in length, with four-year programs comprising less than 25% of EM residency programs in the US.^2^

Approximately 60% of EM program directors (PD) (173/289) from the ACGME database completed a survey created by the Program Requirements Writing Group 3, which found that summed averages for necessary experiences were 41.6 months for three-year programs and 50.7 months for four-year programs. This survey has subsequently been used as justification for the proposed new program requirements, including the 48-month minimum program length. However, the survey did not specifically ask about support for a change from three to four years of training, and it was not designed to examine the impact of any potential changes. The ACGME’s rationale for this change includes concerns about declining board pass rates, potentially attributed to shorter EM shifts and fewer patient encounters during training.^4^ Yet the available published data show that graduates of three- and four-year programs perform similarly in clinical practice and on board pass rates. 5,6

To further explore the perceived challenges and opportunities associated with this change, we surveyed EM PDs on the changes that would be required within their programs and anticipated challenges with the new requirements, and we gauged their support for the proposed requirement of 48 months of training for all EM programs.

## METHODS

We conducted a national cross-sectional survey of EM PDs, or their selected designees (defined as a faculty member delegated by the PD or other residency leadership), from ACGME-accredited EM residency programs in the US over a three-week period in April 2025. After we developed the survey instrument it was piloted for content validity, clarity, and relevance by four members of our educational leadership teams who have experience in program leadership and survey-based research. All feedback was incorporated into the survey, which was approved by our institutional review board as an exempt study. The survey was designed on SurveyMonkey (Momentive Inc., San Mateo, CA) and disseminated to EM PDs through the Council of Residency Directors (CORD) in Emergency Medicine Program Director list-serv. Reminders were sent at one-week intervals for a total of three times. At the time of the study there were 288 PDs in ACGME-accredited EM programs.

The survey (Appendix A) consisted of 11 questions and was divided into three sections: demographic information; curricular changes; and reflection. In the section on proposed curricular changes, participants were asked to assume that the program requirements had been adopted and to answer questions on anticipated changes to their program’s required rotations (62 weeks at primary emergency department [ED], low-resource ED, high-resource ED, low-acuity area, critical care, pediatric intensive care unit, pediatric ED, administration/quality assurance, toxicology/addiction medicine, and emergency medical services). They were then asked about anticipated changes that would be necessary to meet the required structured experiences (non-laboratory diagnostics such as ultrasound, telemedicine, primary assessment and decision-making, airway management, ophthalmologic procedures, acute psychiatric emergencies, sensitive exams, transitions of care, and observation medicine). The final section of the survey included questions on the time needed to adopt the 48-month format, additional resources required (additional funding aside from salary, additional training sites, additional core faculty, additional clinical faculty, more protected time, additional simulation or procedure lab time), and agreement on the proposed changes.

**Population Health Research Capsule**

What do we already know about this issue?

*The ACGME has proposed major changes to emergency medicine (EM) training.*

What was the research question?

*How do program directors view the proposed ACGME changes and what resources are needed to comply?*

What was the major finding of the study?

*33.6% of 3-year and 100% of 4-year programs support the change to 48 months minimum residency training in emergency medicine (P < .001).*

How does this improve population health?

*The study identifies changes that EM programs would need to implement meet new standards, to ensure the future workforce is well-prepared to deliver quality care.*

We summarized categorical survey responses with frequencies and percentages. Chi-square tests were used to evaluate associations. *P*-values < .05 were considered statistically significant. We performed analysis using SAS v9.4 (SAS Institute Inc, Cary, NC).

## RESULTS

A total of 92 respondents completed the survey. However, six were excluded because they did not identify as either a PD or their designee, and their data was not included in the analysis. In total, 86 EM programs were included in the analysis of the survey data for a final response rate of 29.9%. Of the 86 PDs who completed the survey, 72 (83.7%) were from three-year programs and 14 (16.3%) from four-year programs, which is similar to the breakdown of three- and four-year programs currently listed on the Emergency Medicine Residents’ Association Match website.^7^ Most programs were university-based (33, 38.4%), followed by community-based university-affiliated (31, 36%), and community-based (22, 25.6%). Participant programs were geographically representative (Table) of the EM academic community based on Fellowship and Residency Electronic Interactive Database Access geographic regions.^7^

### Curriculum Changes

Of the 86 PDs who responded to the survey, 50 (58%) anticipated needing to make three or more changes to their curricula to meet the required nine structured experiences (Table). This was not significantly different between three- and four-year programs. Of the 86 respondents, 14 (16%) reported already having all the required rotations in the proposed requirements, and 34 (40%) reported that they would need to add three or more rotations. Forty-four respondents (51.2%) indicated that they would be likely to increase their complement of residents, whereas 12 (14%) indicated that they would likely decrease the number of residents. Twenty-five respondents (29.1%) indicated that they were unlikely to change their complement, and five respondents (5.8%) did not answer the question. Most of the programs that would look to expand their complement were currently three-year programs (42/44, 95.5%).

### Time Needed

Forty-nine (57%) PDs indicated readiness for all the changes within a one-year period, while 33 (38.4%) reported they would require more than one year to prepare, 20 (23%) would require two years, and 13 (15%) would require more than three years to prepare.

### Overall Support

Of the PDs of four-year programs, 100% (14/14) supported the change to a minimum 48 months of residency training. However, only 21% (15/72) of three-year PDs supported the change (*P*<.001).

## DISCUSSION

### Structured Experiences

Our survey results indicate that the new program requirements would require a substantial need for curricular revision, which impacts programs differently. For the new “experiences” requirement, only one PD surveyed reported already having all components in place. By contrast, 36% of PDs (31/86) reported that they would probably require one to two changes, and 26% (22/86) would have to make ≥ 5 curricular changes to meet the “experiences” requirements. Curricular revision, including time to pilot, revise, and assess the curricula, is a time-intensive process that can take over a year.

### Required Rotations

The required rotations also pose challenges for programs. While 16% (14/86) reported already having all required rotations, 40% (34/86) of the PDs surveyed reported that they would require ≥ 3 revisions to their rotations. When these new rotations require a new training site, such as adding a low-resource ED, it takes a considerable amount of time to research sites and reach agreements. These external sites can also impact the funding of programs through the Centers for Medicare and Medicaid Services.^8^ Additionally, new rotations may require the addition of new faculty, more faculty development, and institutional agreements that cost money and take time.

### Time Needed

While 57% of the PDs surveyed (49/86) reported readiness for changes in one year, 38% (33/86) reported they would need more than one year to prepare for the new requirements. This affected both three-year (28/72, 39%) and four-year (5/14, 36%) programs. More concerning is that 15% (13/86) of the PDs surveyed anticipated needing more than three years to prepare for the new program requirements. If those programs were truly unable to prepare in the time frame proposed by the Residency Review Committee (RRC) and decided to close their programs, this could have a major impact on the number of trainees in EM. Additionally, should the RRC-EM grant exceptions or extensions to some programs transitioning to a 48-month format, it could create a competitive advantage to those programs in resident recruitment.

### Overall Support

While overall support for the change to 48 months of training was universal for the PDs of four-year programs, there was considerable disagreement among the PDs of three-year programs, with only 15 (22%) supporting the change and 52 (78%) opposing. Our findings of support for a 48-month training requirement mirror a previous study that showed a strong correlation between the current length of a program and its PD’s support for that format.^9^

There has been robust discussion regarding the proposed changes since they were presented. Emergency medicine is not alone in considering lengthening residency training time. Family medicine has also discussed a transition to a 48-month training program, which would entail more study and a gradual transition rather than a sudden turnaround.^10^ The majority of EM program directors surveyed indicated that they would be likely to increase their complements of residents, which could significantly impact the future workforce in EM. Further study is needed to determine how these complement changes may affect the total number of residency spots available in EM each year. More study is needed to fully understand the impacts these changes would have on EM training programs, as well as their impact on the costs involved, especially when considering the lack of evidence to support the extension of training.

## LIMITATIONS

The results of this survey do not include all EM residency programs in the United States, as not all programs are part of CORD, and not all members participate in the list-serv we used to disseminate the survey. Additionally, only 29.9% of programs responded to the survey, creating a significant risk of non-responder bias; however, an appropriate representation of programs both geographically and in terms of length of training was included, which provides support that the data are appropriately representative of the general EM academic community. The narrow three-week window to respond also may have impacted the total number of responses. Finally, we did not collect data on whether the respondent was a program director or their designee, only that they attested to being either the PD or a designee.

## CONCLUSION

Most of the program directors who responded to a survey on the proposed new minimum of 48 months training in emergency medicine were opposed to the change, and a significant minority reported being unprepared to implement the new requirements within one year as proposed by the RRC-EM. If the ACGME does adopt the proposed program requirements in total, multiple years may be required for programs to create new and effective curricula and rotations. More study is needed on the impact of the proposed changes that focuses on the outcomes of graduates. Previous studies have already shown us that graduates of three- and four-year programs perform similarly on the American Board of Emergency Medicine certifying exam and in clinical practice.^5^,^6^ The ACGME should consider a phased roll-out of new requirements to ensure programs have time to thoughtfully and meaningfully adhere to the new requirements in a way that is beneficial to their trainees.

## Supplementary Information



## Figures and Tables

**Table:** Survey results comparing three- and four-year programs by demographic region, program type, time required to implement changes, additional resources required to implement changes, agreement on 48 months of training, total changes needed in experiences, and total changes needed in rotations.

	Frequency (N = 86)	Percentage	3 years (n = 72)		4 years (n = 14)		P-value
What geographic region is your program in (as listed in FRIEDA)?							
East North Central (IL, IN, MI, OH, WI)	15	17.4%	13	18.1%	2	14.3%	
East South Central (AL, KY, MS, TN)	2	2.3%	2	2.8%	0	0.0%	
Mid Atlantic (NJ, NY, PA)	22	25.6%	18	25.0%	4	28.6%	
Mountain (AZ, CO, ID, MT, NM, NV, UT, WY)	3	3.5%	2	2.8%	1	7.1%	
New England (CT, MA, ME, NH, RI, VT)	4	4.7%	2	2.8%	2	14.3%	
Pacific (AK, CA, HI, OR, WA)	5	5.8%	3	4.2%	2	14.3%	
South Atlantic (DC, DE, FL, GA, MD, NC, SC, VA, WV)	16	18.6%	16	22.2%	0	0.0%	
Territory (PR)	1	1.2%	1	1.4%	0	0.0%	
West North Central (IA, KS, MN, MO, ND, NE, SD)	6	7.0%	5	6.9%	1	7.1%	
West South Central (AR, LA, OK, TX)	12	14.0%	10	13.9%	2	14.3%	
What best describes your program?							
Community-based	22	25.6%	19	26.4%	3	21.4%	.27
Community-based, university affiliated	31	36.0%	28	38.9%	3	21.4%	
University based	33	38.4%	25	34.7%	8	57.1%	
Given your current resources, how much time do you feel you would need to create the new rotations and experiences required in the new rules?							
Ready now	6	7.0%	4	5.6%	2	14.3%	
< 1 year	21	24.4%	16	22.2%	5	35.7%	
1 year	22	25.6%	20	27.8%	2	14.3%	
2 years	20	23.3%	15	20.8%	5	35.7%	
3 years	10	11.6%	10	13.9%	0	0.0%	
≥ 4 years	3	3.5%	3	4.2%	0	0.0%	
Missing	4	4.7%	4	5.6%	0	0.0%	
What additional resources would you require to meet the new requirements?*							
Additional funding aside from salary	48	55.8%	45	62.5%	3	21.4%	
Additional training sites	41	47.7%	33	45.8%	8	57.1%	
Additional core faculty	36	41.9%	34	47.2%	2	14.3%	
Additional clinical faculty	20	23.3%	19	26.4%	1	7.1%	
More protected time	41	47.7%	38	52.8%	3	21.4%	
Additional simulation or procedure lab time	36	41.9%	33	45.8%	3	21.4%	
Do you agree with the change to require 48 months of training for all EM programs?							
No	52	60.5%	52	72.2%	0	0.0%	<.001
Yes	29	33.7%	15	20.8%	14	100.0%	
Missing	5	5.8%	5	6.9%	0	0.0%	
Total changes needed in structured experiences							
0	1	1%	1	1%	0	0.0%	.40
1 to 2	31	36%	28	39%	3	21.4%	
3 to 4	28	33%	21	29%	7	50.0%	
≥ 5	22	26%	18	25%	4	28.6%	
Missing	4	5%	4	6%	0	0.0%	
Total changes needed in required rotations							
0	14	16%	11	15%	3	21.4%	.72
1 to 2	34	40%	30	42%	4	28.6%	
3 to 4	24	28%	19	26%	5	35.7%	
≥ 5	10	12%	8	11%	2	14.3%	
Missing	4	5%	4	6%	0	0.0%	
Anticipated changes in resident complement							
Decrease	12	14%	10	13.9%	2	14.3%	<.001
No Change	25	29.1%	15	20.8%	10	71.4%	
Increase	44	51.2%	42	58.3%	2	14.3%	
Missing	5	5.8%	5	6.9%	0	0.0%	

* Not mutually exclusive.

*EM*, emergency medicine; *FRIEDA*, Fellowship and Residency Electronic Interactive Database Access.
