# Supplementary material for: Program Director Perspectives on the Impact of the Proposed 48-Month Emergency Medicine Residency Requirement: A National Survey
Source: West J Emerg Med. 2025 Nov 26;26(6):1504–9. doi: 10.5811/westjem.48359 (PMC12698155; doi:10.5811/westjem.48359)
Supplement: Supplementary file 1 [file wjem-26-1504-s001.docx]

Appendix A. Survey Questionnaire for Program Director Perspectives on the Impact of ACGME’s 48-Month Emergency Medicine Residency Requirement

**Section 1: Demographic Data**

In order to prevent duplication of data while providing the maximal confidentiality possible, we ask that only the residency program director, (or a faculty member delegated by the program director or other residency leadership) complete this survey.

1. Are you a residency Program Director or completing on their behalf as outlined above?

1. Yes
2. No

2. What is the current length of training in your program?

1. 3 years
2. 4 years

3. What is the current total complement of residents your categorial program is approved for?

Number slider from 16 to 200

4. What geographic region is your program in (as listed in FRIEDA)?

New England (CT, MA, ME, NH, RI, VT), Mid Atlantic (NJ, NY, PA), East North Central (IL, IN, MI, OH, WI), West North Central (IA, KS, MN, MO, ND, NE, SD), South Atlantic (DC, DE, FL, GA, MD, NC, SC, VA, WV), East South Central (AL, KY, MS, TN), West South Central (AR, LA, OK, TX), Mountain (AZ, CO, ID, MT, NM, NV, UT, WY), Pacific (AK, CA, HI, OR, WA), Territory (PR)

5. What best describes your program?

Community-based, Community-based university affiliated, University based.

**Section 2: Curricular changes**

For the following, assume that the 2027 Proposed Rules are adopted in whole.

6. What do you anticipate your complement of residents would become?

Number slider from 16 to 200

7. For the following proposed required rotations please indicate if they already exist, need modification, or do not exist in your program:

| Rotation | Already have, no modification needed | Already have, but needs modification | Do not currently have |
| --- | --- | --- | --- |
| 62 weeks at Primary ED |  |  |  |
| Low Resource ED |  |  |  |
| High Resource ED |  |  |  |
| Low-Acuity Area |  |  |  |
| Critical Care |  |  |  |
| PICU |  |  |  |
| Peds ED |  |  |  |
| Administration/ Quality Assurance |  |  |  |
| Toxicology / Addiction Medicine |  |  |  |
| EMS |  |  |  |

8. For the following proposed required required experiences please indicate if they already exist, need modification, or do not exist in your program:

| Experiences | Already have, no modification needed | Already have, but needs modification | Do not currently have |
| --- | --- | --- | --- |
| Non-laboratory diagnostics (ex. Ultrasound) |  |  |  |
| Telemedicine |  |  |  |
| Primary assessment and decision making |  |  |  |
| Airway Management |  |  |  |
| Ophthalmologic Procedures |  |  |  |
| Acute Psychiatric Emergencies |  |  |  |
| Sensitive Exams |  |  |  |
| Transitions of Care |  |  |  |
| Observation Medicine |  |  |  |

**Section 3: Reflection**

9. Given your current resources, how much time do you feel you would need to create the new rotations and experiences required in the new rules?

Ready now, <1 year, 1 year, 2 years, 3 years, 4 or more years

10. What additional resources would you require to meet the new requirements?

Additional funding aside from salary, Additional training sites, Additional Core Faculty, Additional Clinical Faculty, More protected time, Additional simulation or procedure lab time

11. Do you agree with the change to require 48 months of training for all EM programs?

Yes, No
